# ANATOMOTOPOGRAPHIC STUDY OF THE MARTIN-GRUBER ANASTOMOSIS

**DOI:** 10.1590/1413-785220253305e290615

**Published:** 2025-09-22

**Authors:** Edie Benedito Caetano, Cristina Schmitt H Cavalheiro, Núbia dos Reis Sampaio, Pedro Mariano Coelho, Luiz Angelo Vieira, Julio Cesar Gali

**Affiliations:** 1 Pontificia Universidade Católica de São Paulo (PUC), Faculdade de Ciencias Medicas e da Saúde de Sorocaba, São Paulo, SP, Brazil.

**Keywords:** Peripheral Nervous System, Dissection, Surgical Procedures, Sistema Nervoso Periférico, Dissecação, Procedimentos Operatórios

## Abstract

**Objective:**

To create, through anatomical dissections, a map of the location of the Martin-Gruber anastomosis (MGA) in the forearms of cadavers.

**Methods:**

One hundred forearms from 50 adult cadavers were used in this study. Dissection was performed through a median incision in the forearm and distal third of the arm. Lines between the humeral epicondyles (interepicondylar) and between the styloid processes of the radius and ulna (interstyloidea) were used as reference points for the topographic location of the anastomoses, and the forearms were divided into proximal, middle and distal thirds.

**Results:**

MGA was present in 27 forearms (27%). In four limbs (14.8%) the nerve fascicles originated from the median nerve proximal to the interepicondylar line. In two limbs (7.4%), at the level of the interepicondylar line and, in 21 of these (77.7%), they were found distal to this line. In 17 limbs (62.9%), the anastomosis occurred in the proximal third of the forearm, in eight limbs (29.6%), the anastomosis occurred in the middle third of the forearm and, in two limbs (7.4%), the anastomosis occurred with the ulnar nerve it occurred in the distal third of the forearm.

**Conclusion:**

Despite the great variation in their location, most anastomoses were found distal to the interepicondylar line, especially in the proximal third of the forearm. ***Level of Evidence IV; Case Series.***

## RESUMO

**Objetivo:**

Criar, através de dissecações anatômicas, um mapa do local da ocorrência da anastomose de Martin-Gruber (AMG) em antebraços de cadáveres.

**Métodos:**

Cem antebraços de 50 cadáveres adultos foram utilizados neste estudo. A dissecação foi realizada por uma incisão mediana no antebraço e no terço distal do braço. Linhas entre os epicôndilos umerais (interepicondilar) e entre os processos estiloides do rádio e ulna (interestiloidea) foram usadas como pontos de referência para a localização das anastomoses e os antebraços foram divididos em terços proximal, médio e distal.

**Resultados:**

A AMG esteve presente em 27 antebraços (27%). Em quatro membros (14,8%) os fascículos nervosos se originaram do nervo mediano, proximalmente à linha interepicondilar. Em dois membros (7,4%) a origem deu-se no nível da linha interepicondilar e, em 21 destes (77,7%), foram encontrados distais à esta linha. Em 17 membros (62,9%) a anastomose aconteceu no terço proximal do antebraço, em oito membros (29,6%), a anastomose ocorreu no terço médio do antebraço e, em dois membros (7,4%), a anastomose com o nervo ulnar deu-se no terço distal do antebraço.

**Conclusão:**

Apesar da grande variação de sua localização, a maioria das anastomoses foi encontrada distalmente à linha interepicondilar, especialmente no terço proximal do antebraço. ***Nível de Evidência IV; Série de Casos.***

## INTRODUCTION

There are several possibilities for nerve communication in the upper limbs. These anatomical variations, when occurring between the median and ulnar nerves, are given different names according to their configurations. When the fascicles of the ulnar nerve branch toward the median nerve, they are known as Marinaci or reverse Martin-Gruber anastomoses.^1^ They are called Cannieu and Riché anastomoses when the thenar motor branch of the median nerve anastomoses with the deep motor branch of the ulnar nerve^2,3^ and Berretini anastomoses when they occur between the sensory branches in the palm of the hand.^4,5^ When communication occurs at the point where the fascicles of the median nerve are directed toward the ulnar nerve in the forearm, it is known as Martin-Gruber anastomosis (MGA), in honor of the studies conducted by Martin and later by Gruber.^6,7^ This anastomosis is responsible for creating variations in innervation, especially in the intrinsic muscles of the hand.^8,9^

Due to these variations in the pattern of innervation, compressive syndromes or isolated lesions of the median or ulnar nerve may have clinical presentations that differ from the classic patterns described in anatomy textbooks.^10^ Anatomical and electrophysiological studies suggest that these communications have relevant clinical and surgical implications,^11–13^ making their knowledge of great importance for the diagnosis, treatment, and performance of various procedures in the elbow and forearm regions.^11,12^ The objective of this study was to create a topographic map of the sites of AMG occurrence to assist surgical procedures performed on the elbow and forearm.

## MATERIALS AND METHODS

This research was approved by our institution's Ethics Committee under number 43267715.2.0000.5373. One hundred forearms from 50 adult cadavers were dissected for this scientific study. Forty-six bodies were male and four were female. The age range of the individuals varied from 28 to 77 years. Thirty-eight specimens were prepared using a 10% formaldehyde and glycerin solution. As the other 12 had recently passed away, they did not require this preparation. Forearms deformed by trauma or those with malformations were excluded from this study.

The dissection was performed through a median incision in the distal third of the arm and throughout the forearm. The two flaps created, including the skin and subcutaneous tissue, were reflected radially and ulnarly, respectively, and the same was done with the forearm fascia to expose all the muscles in this region. The interepicondylar line (between the medial and lateral epicondyles of the humerus) and the interstyloid line (between the styloid processes of the radius and ulna) were used as reference points to measure the topographic location of the AMG.

A Keeler magnifying glass with 2.5x magnification was used to improve visualization of the anatomical structures. The length of the communication between the median and ulnar nerves was measured with a caliper by two researchers. It was considered the average between the measurements obtained by the two individuals. We drew a line between the medial and lateral humeral epicondyles (interepicondylar) and another between the styloid processes of the radius and ulna (interstyloid), which were used as reference points for measuring the topographic location of the AMG. The region between the two lines was divided into proximal, middle, and distal thirds. All muscles of the forearm were dissected to analyze their innervation and the presence of nerve communication between the median and ulnar nerves. All Martin-Gruber anastomoses found were recorded and photographed.

## RESULTS

**Figure 1:**
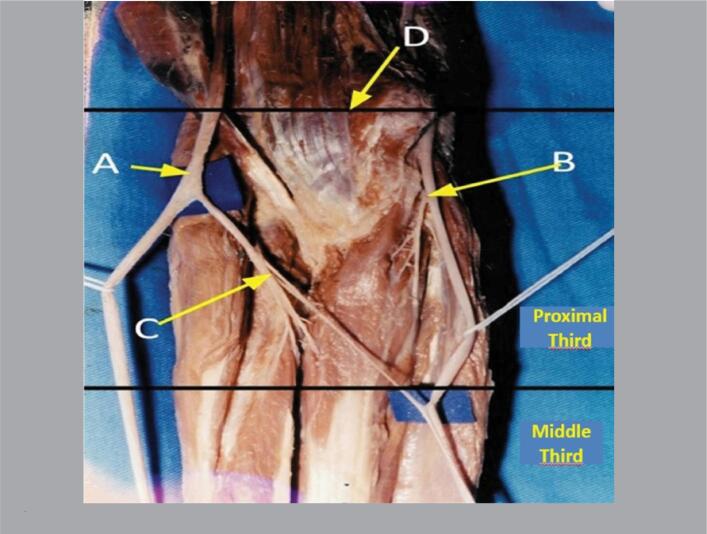
Anterior view of the right forearm divided into its proximal, middle, and distal thirds, where we can see the median nerve (A), ulnar nerve (B), and nerve fascicles detached from the median nerve (C) proximal to the interepicondylar line of the humerus (D).

Source: Authors.

**Figure 2:**
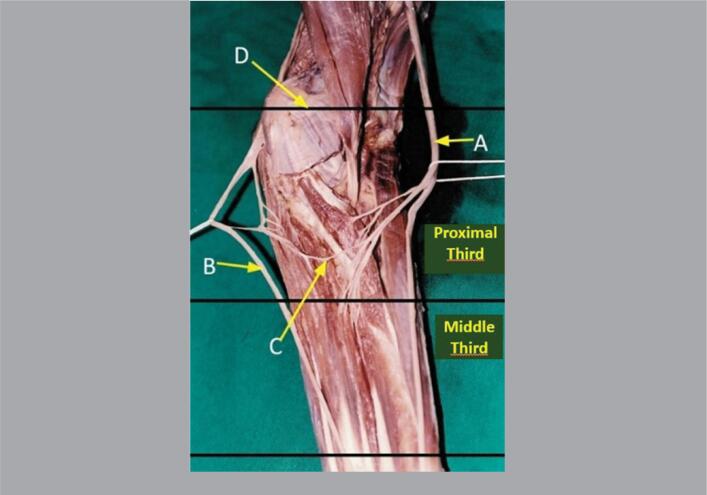
Anterior view of a right forearm divided into its proximal, middle, and distal thirds, where we can see the median nerve (A), ulnar nerve (B), nerve fascicles detached from the median nerve (C) at the level of the interepicondylar line of the humerus (D), and the anastomosis occurred in the middle third of the forearm.

Source: Authors.

**Figure 3:**
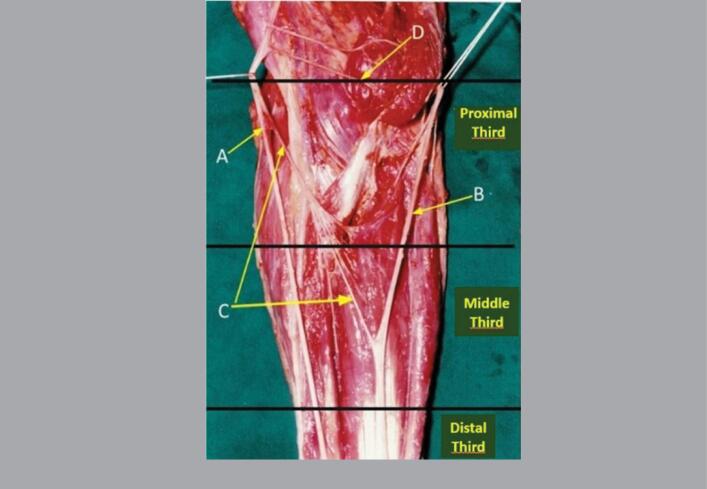
Anterior view of a left forearm divided into its proximal, middle, and distal thirds, where we can see the median nerve (A), ulnar nerve (B), nerve fascicles detached from the median nerve (C) distally to the interepicondylar line (D), and the anastomosis in the proximal third of the forearm.

Source: Authors.

**Figure 4:**
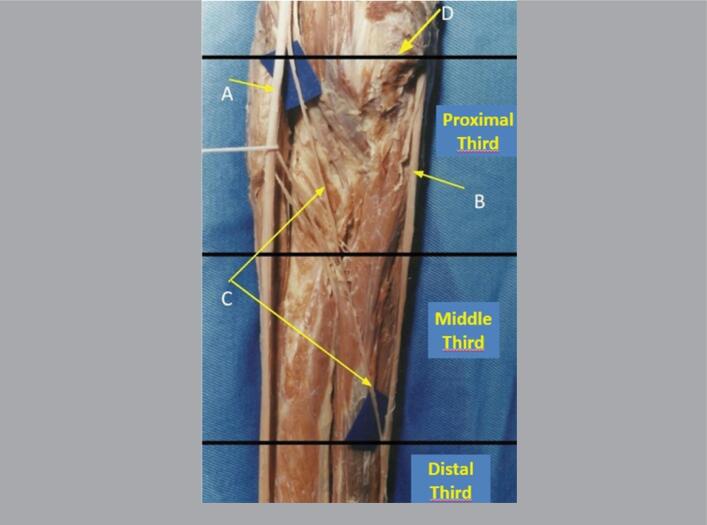
Anterior view of a left forearm divided into its proximal, middle, and distal thirds, where we can see the median nerve (A), ulnar nerve (B), nerve fascicles detached from the median nerve (C) distally to the interepicondylar line (D), and the anastomosis performed in the middle third of the forearm.

Source: Authors.

AMG was detected in 27 of the 100 dissected forearms (27%). Of these, 13 presented nerve damage in the right upper limb (48.1%) and 11 in the left upper limb (40.7%). Bilateral involvement was observed in three cadavers (11.1%). With regard to the topographical location of the AMG, we observed that in four members (14.8%), the nerve fascicles were detached from the median nerve proximally to the interepicondylar line of the humerus (Figure 1), with measurements ranging from 2 to 12 mm (mean 4 mm). In two members, this occurred at the level of the interepicondylar line (7.40%) (Figure 2). In twenty-one members (77.7%), the nerve fascicles were detached from the median nerve distally to the interepicondylar line (Figures 3 and 4). In these, the length of nerve communication ranged from 45 to 227 mm (average 74 mm).

In five members (18.5%), the nerve connection was recorded inside the deep flexor muscle mass of the fingers, all of which were found in the proximal third of the forearm. In 17 members (62.9%), the anastomosis occurred in the proximal third of the forearm (Figure 3), in eight limbs (29.6%), anastomosis occurred in the middle third of the forearm (Figure 2), and in two limbs (7.4%), anastomosis with the ulnar nerve occurred in the distal third of the forearm (Figure 5). The most distal communication identified in this study was 75 mm proximal to the intersutural line.

## DISCUSSION

The main finding of our study was that most Martin-Gruber anastomoses were found distal to the interepicondylar line, in the proximal third of the forearm, and that there was a wide variation in their location. The incidence of nerve communication between the median and ulnar nerves in the forearm varies in the literature. In this study, we found AMG in 27% of dissected forearms, while in other studies, the incidence of nerve communication ranged from 7.8 to 32%.^7,11-19,20^

In our series, 48.1% of nerve injuries occurred in the right upper limb, 40.7% in the left upper limb, and 11.1% were bilateral. In the study by Kaur et al.,^21^ anastomoses occurred on the right side in 42.8% of specimens, on the left side in 28.5% of cases, and were bilateral in 28.5% of limbs. Roy et al.^22^ in a meta-analysis, found that AMG was most commonly found unilaterally (66.8%), with 15.7% occurring on the right side. For these authors, the length of this nerve communication was 41.55 mm ± 13.77 mm, whereas in our assessment, the measurement varied significantly, ranging from 45 to 227 mm, with an average of 74 mm.

Electroneuromyographic studies also evaluated AMG. Hefny et al.^23^ evaluated 280 forearms from 140 healthy Egyptians with a mean age of 36.5 years (range, 23-58 years) and found nerve connections in 20% of the individuals analyzed. Bilateral involvement occurred in 4.28% of these cases and was more frequent on the right side. In the study by Sur et al.^24^ the AMG was found in 21.4% of 140 Indian individuals. Bilateral involvement occurred in 46.6% of the people evaluated, with AMG identified in the right forearm in 26.6% of cases and in the left forearm in 26.6% of cases. However, studies using electrical stimulation may show discrepancies due to minor variations in technique, such as placing the electrodes at different depths, making them less accurate.

In another article, Gans & Alfen^25^ described a case of AMG in which the study was performed by ultrasound of the median and ulnar nerves, from the armpit to the palm. The anastomosis occurred in the proximal forearm, where the median nerve branch detached shortly after emerging, below the pronator teres muscle, to join the ulnar nerve 50 mm more distally. Although there are subsidiary tests to assess the existence of AMG, we believe that the study conducted through anatomical dissections is still of great importance and accuracy.

Upon reviewing the articles published on AMG, we observed that the authors evaluated its incidence, the nature of the nerve fibers, and its classification without, however, studying the topographical location of the anastomosis. Therefore, our research did not find any studies in the English-language literature that analyzed the location of AMG occurrence, so that we could not compare our results.

## CONCLUSION

Although there is considerable variation in the location of occurrence, most Martin-Gruber anastomoses were found distal to the interepicondylar line, in the proximal third of the forearm.
